# Marginalising dyslexic researchers is bad for science

**DOI:** 10.7554/eLife.93980

**Published:** 2023-12-15

**Authors:** Helen Taylor, Arash Zaghi, Sara Rankin

**Affiliations:** 1 Hunter Centre for Entrepreneurship, University of Strathclyde Glasgow United Kingdom; 2 Department of Civil and Environmental Engineering, University of Connecticut Storrs United States; 3 National Heart & Lung Institute, Faculty of Medicine, Imperial College London London United Kingdom

**Keywords:** dyslexia, neurodiversity, evolution, learning, exploartive learning, AI assitive tools

## Abstract

Communication in the sciences is often based on text, which places researchers with dyslexia at a disadvantage. However, this means that science is missing out on the original insights and specific strengths in exploration that dyslexic researchers bring to their disciplines. Here we discuss how the scientific community can address the challenges that dyslexic researchers face, and how science stands to benefit as a result. We discuss this in the context of a new theoretical framework proposing the existence of complementary learning strategies that could play a key role in scientific progress, particularly with regard to accelerating innovation.

## What is dyslexia?

From the traditional or medical model perspective, developmental dyslexia is defined as a disorder “in children who, despite conventional classroom experience, fail to attain the language skills of reading, writing and spelling, commensurate with their intellectual abilities” (World Federation of Neurology, 1968). Co-occurring difficulties may be seen in aspects of motor coordination, concentration, rote learning and time management.

Dyslexia is the most commonly recognized ‘specific learning difficulty’, which also includes dyspraxia (also known as developmental co-ordination disorder), dyscalculia, dysgraphia and attention deficit hyperactivity disorder (ADHD). The prevalence for dyslexia spans from around 5–20%, with the range reflecting differences in writing systems, diagnostic assessments and the continuous distribution of dyslexia indicators in the general population (Wagner et al., 2020). Dyslexia is also a complex trait involving multiple genes with a heritability of at least 60% (Paracchini et al., 2016). These factors, along with its high prevalence, suggest that rather than a disorder, this form of cognition may have an evolutionary advantage.

Indeed, the learning difficulties associated with dyslexia may not reflect the whole picture. Research published in the 1970s and 1980s already suggested that dyslexia may be associated with considerable advantages in mechanical, visual-spatial and creative endeavours (see, for example, Critchley, 1970; Geschwind, 1982). The pioneering behavioural neurologist Norman Geschwind described dyslexia as a “pathology of superiority”, suggesting that differences in the brain “…that have led to the disability of dyslexia in certain literate societies also determine superiority in the same brains”Indeed, Geschwind’s insight that the difficulties exist as a trade-off to important strengths turns out to be key importance, as we will return to below.

## Attainment levels in schools

Students with dyslexia and other specific learning differences have tended to underperform at school relative to their peers (Table 1). In England, for example, students sit exams, called GCSEs, when they are 16 years old: around a fifth of students who have a special educational needs support (but not a education and healthcare plan) receive grade 5 or above for English and maths in these exams, compared with around half for students who do not have this support (Table 1). Similar attainment gaps have been seen in other studies (see, for example, Nunes et al., 2017).

**Table 1.** GCSE results from state schools in England between 2018/19 and 2022/23. Percentage of pupils achieving grade 5 or above in English and maths for pupils with no special educational needs (SEN) and pupils with SEN support. (UK Government, 2023).

	Percentage of pupils achieving grade 5 or above in English and Mathematics				
	2018/19	2019/20	2020/21	2021/22	2022/23
Pupils with no SEN	48.2	55.8	58.0	55.9	50.7
Pupils with SEN support	16.8	20.5	22.2	22.5	20.5

GCSE results for state schools in England.

Consistent with low attainment levels in schools, data from a report commissioned by the Royal Society in 2019 indicates that only 5% of students studying a STEM (science, technology, engineering and mathematics) subject at university in the UK have a specific learning difference, with this number dropping to 0.9% for academics working in STEM subjects (Joice and Tetlow, 2021). We contend that the low percentage of students with specific learning differences studying STEM subjects at UK universities is due to low attainment levels in national exams (such as GCSEs), which deters or prevents them from studying STEM subjects at university. The even lower percentage of STEM academics with a specific learning difference is likely due to a combination of low absolute numbers and low rates of disclosure. The latter may be due to the negative stigma associated with having a specific learning difference, or the fact that many individuals have never had a formal diagnosis. Dyslexic academics are not generally offered support in the context of their academic careers, so for most there is no point in either getting a late diagnosis or disclosing a diagnosis.

We suggest that an overemphasis on text-based communications for the assessment of STEM subjects in schools (via exams) and the evaluation of scientific outputs in higher education (via papers and grants) has inadvertently disadvantaged the dyslexic community in these disciplines. However, there are a myriad of ways of communicating, storing and sharing knowledge and data that are not text-based. If we diversify the ways that STEM subjects are communicated and assessed, we will make them more neuro-inclusive (Box 1).

Box 1. **Changes that would make STEM more inclusive**

*Academic writing support*: scientific journals could provide writing and editing support for dyslexic scientists.

*Flexible assessment strategies:* Universities and academic institutions could incorporate alternative assessment methods that provide diverse ways for dyslexic individuals to demonstrate their knowledge and understanding. This could include oral exams, practical demonstrations, podcasts, mind-maps and project-based assessments, which can accommodate different learning styles and cognitive strengths.

*Administrative support*: dyslexic individuals may benefit from assistance to manage and co-ordinate the diverse roles of an academic, including teaching, research, tutoring, mentoring, EDI activities, outreach, attending and organising internal and external meetings and conferences.

*Provision of and training in assistive technologies*: specific tools, such as mind-mapping software, allows users to visually organise and map information, thoughts and projects, while AI-powered language models could be used to fine-tune written information.

*Recognition and acceptance* of different forms of communication as valid research outputs, for example, making recorded lectures publicly available.

*Expanding research communication*: scientific journals could provide more options for communicating research, which should further be recognised in the context of academic assessment, career progression and grant applications.

## Global exploratory learning as a new framework for understanding dyslexia

Many of the challenges dyslexics face arise from modern-day assumptions that people communicate and learn through reading, writing and memorising existing knowledge. However, this approach is called into question if we look back over the human past.

Behaviourally modern humans have existed for at least 50,000 years, but writing – first invented around 5,000 years ago – has only been used widely in the last century (Englund, 2004). This challenges the notion that difficulties in writing signifies a deficit, highlighting it as a unique cultural invention with no evolutionary imperative. In fact, writing is possibly the only cultural tool where we equate difficulty with deficit.

Literacy problems may also reflect a more generalised difficulty in learning skills until they become automatic (Nicolson and Fawcett, 1990). This is particularly problematic in western education, where learning typically focuses on exploitation of existing knowledge, such as rote learning and memorisation. However, it is well-known that learning occurs on a continuum, ranging from exploitation of existing knowledge to exploration of unknown options (March, 1991; Holland, 1992). Balancing this trade-off is key to maintaining and improving the knowledge we need to adapt and survive.

Researchers – including one of the present authors (Helen Taylor) – have suggested that dyslexia might reflect a specialization at the latter end of this continuum: a more global-level exploratory learning (Taylor and Vestergaard, 2022). This is manifested in strengths such as generating new knowledge through discovery or invention – precisely what is needed in academia and biotechnology.

But in the current academic system, career advancement is primarily based on the quantity of written output which favours iteration over paradigm shifts in understanding. Academia offers greater support to specialists in subject-specific domains over individuals who explore across disciplines. Thus, although academic research is ostensibly explorative (i.e., about furthering knowledge), the way highly explorative academics work is not aligned with the current academic system.

In general, the more variable and complex the environment is, the more we need to learn through exploration. Exploratory learning involves searching the environment and memory for relevant information, enabling us to construct internal representations of the world to help predict what might happen in less familiar situations. In dyslexic people, such representations are reported as being particularly ‘global’ (i.e., multidimensional, dynamic and often highly visual). Thomas West, who spent decades interviewing dyslexic scientists, described their ability to build complex mental models or to have strikingly unusual insights, and their potential “to look over the horizon” or to see patterns in nature that others do not see (West, 1991; West, 2010; West, 2017).

Global exploratory learning can also be understood as an aptitude for understanding complexity and the dynamics of complex adaptive systems. Such systems consist of networks of interdependencies and are more than the sum of their parts; therefore, they need to be understood holistically and contextually. This can be reflected, for example, as an aptitude for exploring potential interactions in an ecosystem or inside a cell, and understanding how these might change through time, or recognition of fundamental (global) patterns. Such a way of thinking may enable researchers to translate insights and solutions between different domains of knowledge. The importance of this kind ofthinking approach is increasingly recognised as critical to solving many modern-day problems in science (Arnold and Wade, 2015; Dominici, 2012).

The global exploratory learning specialization in dyslexics may be understood in the context of a recent theory called “complementary cognition” (Taylor et al., 2022). This theory proposes that humans evolved to specialise in different but complementary learning strategies, which, through collaboration, enable us to co-create the knowledge we need to adapt and survive (‘collaborative learning’). Collaborative learning can be thought of as an interactive process whereby group knowledge is continually revised, involving global exploration, local refinement and consolidation, so that we collaboratively update our understanding of the world.

Explorative and collaborative learning can also have important consequences for scientific progress. Underexploring diminishes our capacity to update knowledge and advance scientific understanding (March, 1991). Indeed, recent reports suggest a decline in disruptive scientific contributions, indicating an under-exploration in the scientific community (Park et al., 2023). In contrast, nurturing global explorative learning and supporting collaborative learning could confer enormous benefits. For example, exploring globally first can avoid getting trapped in a local optimum, helping to identify out-dated paradigms. By building representations of the broader problem space, global explorers may help guide which options should be developed in greater detail.

Although research is still ongoing to understand dyslexia, exploratory and collaborative learning, increased recognition of the theoretical framework may facilitate team building and develop understanding of complementary strengths to build better scientific collaborations. An important first step, however, is to make STEM more accessible and inclusive.

## Cognitive diversity in business

In March 2023, the UK BioIndustry Association published its first report on diversity and inclusion, based on surveys completed by over 1,200 individuals from 30 companies (UK BioIndustry Association, 2023). Around 11% of non-management employees and 13% of individuals in management positions declared a specific learning difference. This result is in stark contrast to the 0.9% of STEM academics in the UK disclosing a specific learning difference. Does this reflect the research culture and lack of psychological safety in higher education institutions? Or does it highlight real barriers to career progression for dyslexic academics, leading them to leave academia in favour of joining the biotech industry or alternative career paths that are more neuro-inclusive?

Many businesses that employ STEM graduates, including AstraZeneca, Government Communications Headquarters, Syngenta, Microsoft, IBM, Google, Ernst & Young, GSK and BenevolentAI, are now recognising the value of recruiting individuals with specific learning differences to increase the cognitive diversity of their workforce and drive innovation in their sectors. They have set up employee networks and continue making the workplace more neuro-inclusive, something that is not currently happening at the same pace in academia.

## The problem with text-based academic publications

Today’s students have a vast array of resources to access STEM learning, from picture books and computer games to virtual reality, films, animations, audio files, and interactive gamification. They can also explore STEM centres and science festivals. Such multi-modal and interactive learning resources cater to the diverse learning preferences of students, including dyslexic students. However, in the formal education setting of classrooms, the dominant pedagogical approach still heavily relies on traditional textbooks. Furthermore, STEM assessments largely depend on literacy skills (reading, writing and comprehension), inadvertently creating a challenge for dyslexic students whose strengths may lie outside of text-based learning.

Moreover, for research to be ‘recognized’, it has to be published in a scientific journal, with data presented graphically alongside a text-based narrative, and with each journal dictating their own, mostly rigid format. This can prove to be a challenge for dyslexic scientists, as for many, their understanding of scientific problems is systems based over multiple levels that often span different disciplines. Thus, translating the complexity of these thoughts, which are often viewed as networks and patterns, into linear one-dimensional text is challenging.

Dyslexic individuals frequently have a penchant for visual and oral communication, which presents an opportunity to make scientific publishing more inclusive. The digitisation of scientific journals has already enabled researchers to submit 3D images, videos and links to data sets. Indeed, the visualisation of big data has necessitated a move away from traditional graphical presentation of data. Publishing companies that present graphical abstracts alongside traditional text-based abstracts are taking one step towards making science more accessible to dyslexics.

Centuries ago, scientists shared their discoveries and inventions through demonstration lectures in places like the Royal Society. And even though research is nowadays still presented at scientific conferences (live and increasingly online), these presentations are not usually accepted research outputs. Scientific success is still predominantly measured through text-based publications that occur months to years later.

## Assistive technologies can help make STEM more accessible

At the vanguard of technological revolution is the galloping advancement of artificial intelligence (AI) and assistive technologies, which present a pivotal opportunity for the dyslexic community. Especially important are ‘large language models’, which have the potential to dramatically shift traditional academic paradigms. Harnessing the potential of such models to supplement writing represents a crucial juncture for dyslexics.

Therefore, academia’s evaluation paradigms should recentre and prioritize originality and innovation over the ability to write articulate narratives. This would ensure that academics are assessed on the basis of the substance of their academic contributions rather than prowess in ornate writing. This would prompt more inclusivity in academic evaluations, fostering an environment where diverse thinking and unconventional problem-solving approaches thrive.

The growing popularity and ability of AI technologies brings us closer to a future where the translation of research findings across various modalities becomes seamlessly efficient. A future in which textual content can be intuitively refashioned to cater to individual consumer preferences or transformed into visual mediums no longer seems far-fetched.

These forthcoming possibilities emphasize the necessity for academic publishers to embrace AI’s transformative power, accelerating diversification in academic research dissemination methods. This embrace necessitates a fundamental shift in academia’s structure, prompting broader, more inclusive thinking. By making academic research more accessible it will be amplified, casting a wider net in reaching larger audience demographics. By enabling a larger spectrum of non-traditional students to contribute, such developments will inevitably spawn an era of prolific STEM creativity, thereby fostering a collectively rich intellectual growth across the academic discourse.

## Conclusions

Our examination of STEM education, knowledge assessment and communication reveals a bias toward traditional text-based approaches and abilities related to exploitation. This has inadvertently marginalised the dyslexic community who, according to recent research, have strengths more related to broad exploration such as innovative, non-linear, and systems thinking. Consequently, academia may be restricting the pace of scientific discovery. We therefore underscore the urgent need to adopt inclusive communication practices and redefine our evaluation metrics to foster a more creative STEM academic environment. Finally, we propose that the key to scientific progress in terms of accelerating innovation, is establishing collaborative teams that leverage complementary learning strategies (relating to the degree of exploration and exploitation).
